# Crucial Role of Hyaluronan in Neointimal Formation after Vascular Injury

**DOI:** 10.1371/journal.pone.0058760

**Published:** 2013-03-06

**Authors:** Yuichiro Kashima, Masafumi Takahashi, Yuji Shiba, Naoki Itano, Atsushi Izawa, Jun Koyama, Jun Nakayama, Shun'ichiro Taniguchi, Koji Kimata, Uichi Ikeda

**Affiliations:** 1 Department of Cardiovascular Medicine, Shinshu University Graduate School of Medicine, Matsumoto, Japan; 2 Division of Bioimaging Sciences, Center for Molecular Medicine, Jichi Medical University, Tochigi, Japan; 3 Department of Molecular Biosciences, Faculty of Life Sciences, Kyoto Sangyo University, Kyoto, Japan; 4 Department of Molecular Pathology, Shinshu University Graduate School of Medicine, Matsumoto, Japan; 5 Department of Molecular Oncology, Shinshu University Graduate School of Medicine, Matsumoto, Japan; 6 Research Complex for the Medicine Frontiers, Aich Medical University, Aichi, Japan; Osaka University Graduate School of Medicine, Japan

## Abstract

**Background:**

Hyaluronan (HA) is a primary component of the extracellular matrix of cells, and it is involved in the pathogenesis of atherosclerosis. The purpose of this study was to investigate the role of HA in neointimal formation after vascular injury and determine its tissue-specific role in vascular smooth muscle cells (VSMCs) by using a cre-lox conditional transgenic (cTg) strategy.

**Methods and Results:**

HA was found to be expressed in neointimal lesions in humans with atherosclerosis and after wire-mediated vascular injury in mice. Inhibition of HA synthesis using 4-methylumbelliferone markedly inhibited neointimal formation after injury. *In vitro* experiments revealed that low-molecular-weight HA (LMW-HA) induced VSMC activation, including migration, proliferation, and production of inflammatory cytokines, and reactive oxygen species (ROS). The migration and proliferation of VSMCs were mediated by the CD44/RhoA and CD44/ERK1/2 pathways, respectively. Because HA synthase 2 (HAS2) is predominantly expressed in injured arteries, we generated cTg mice that overexpress the murine *HAS2* gene specifically in VSMCs (cHAS2/CreSM22α mice) and showed that HA overexpression markedly enhanced neointimal formation after cuff-mediated vascular injury. Further, HA-overexpressing VSMCs isolated from cHAS2/CreSM22α mice showed augmented migration, proliferation, and production of inflammatory cytokines and ROS.

**Conclusion:**

VSMC-derived HA promotes neointimal formation after vascular injury, and HA may be a potential therapeutic target for cardiovascular disease.

## Introduction

Neointimal formation after vascular injury is the pathological basis of the restenosis that occurs after revascularization procedures such as percutaneous coronary intervention (PCI). The vascular smooth muscle cells (VSMCs) are the main factors involved in vascular wall remodeling after such injury, and it is currently accepted that neointimal formation after injury involves migration of medial VSMCs toward the lumen, where they proliferate and secrete extracellular matrix (ECM) proteins [1]. An accumulating body of evidence suggests that the interaction of ECM proteins with VSMCs plays a crucial role in the processes of neointimal formation after vascular injury [2], although the underlying mechanisms are not completely understood.

Hyaluronan (also known as hyaluronic acid, HA) is a large, nonsulfated glycosaminoglycan that is ubiquitously present in the ECM of all vertebrates. Mammalian HA is synthesized by 3 HA synthases (HAS): HAS1, HAS2, and HAS3 [3], [4]. The physiological and pathological effects of HA have been shown to depend on the chain length [4], [5]. High-molecular-weight HA (HMW-HA >500 kDa) is the predominant isoform under physiological conditions. However, it fragments during inflammation and tissue injury, and these HA fragments (i.e., low-molecular-weight HA [LMW-HA], <500 kDa) exert angiogenic and proinflammatory effects. One study showed that HA overexpression in the vascular tunica media promoted the development of atherosclerosis in apolipoprotein E-deficient (apoE^–/–^) mice [6]. On the other hand, a recent investigation showed that inhibition of HA synthesis by treatment with 4-methylumbelliferone (4-MU) accelerated atherosclerosis in Western diet-fed apoE^–/–^ mice [7]. Increased production of HA in balloon-injured rat carotid arteries was also reported [8]. However, the contribution of HA to neointimal formation after vascular injury is not completely understood. In the present study, we examined the role played by HA in 2 types of vascular injury models *in vivo* and studied the effect of LMW-HA and HMW-HA on the migration and proliferation of VSMCs *in vitro*. In particular, since little is known about the tissue-specific role of HA, we generated conditional transgenic (cTg) mice that overexpress murine HAS2 specifically in VSMCs and examined the role of VSMC-derived HA. The study findings demonstrate the crucial role played by HA in vascular injury and suggest that HA is a potential therapeutic target for cardiovascular disease.

## Methods

### Ethics statement

All animal experimental procedures and protocols were approved by the Animal Experiment Committee of Shinshu University and were performed according to the Institutional ethical guidelines of for animal experiments. Human autopsy samples were obtained with written informed consent from the families and analyzed under the approval from the Ethics Committees of Shinshu University.

### Animals and human samples

**Figure 1:**
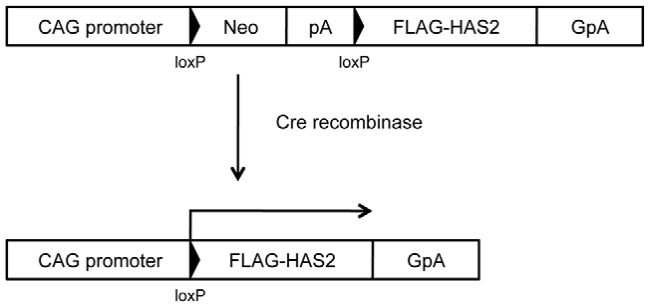
HAS2 conditional Tg (cHAS2) mice. FLAG-tagged murine HAS2 cDNA was positioned downstream of the transgene unit, including the CAG promoter, a loxP sequence, the neo-resistance gene (*Neo*), the SV40 poly(A) signal (pA), and a second loxP sequence. On recognition of the loxP site, Cre recombinase deletes the *Neo* cassette along with one of the loxP sequences and then joins the CAG promoter and HAS2 cDNA, leading to expression of HAS2 mRNA.

Mice were anesthetized by intraperitoneal injection of the avertin (0.75 mg/g body weight) and euthanized by cervical dislocation prior to tissue collection. We used HAS2 cTg mice (cHAS2 mice) showing Cre recombinase-dependent expression of HAS2, which have been described previously (Fig. 1) [9], [10]. CreSM22α transgenic mice that overexpress Cre recombinase under the control of the SM22a promoter were kindly provided by Dr. Joseph M. Miano (University of Rochester, NY) [11], [12]. cHAS2/CreSM22α mice that overexpress HAS2 in VSMCs were generated by crossing cHAS2 and CreSM22α mice. Other wild-type mice were purchased from Japan SLC Inc. (Hamamatsu, Japan). All mice used in this study had a C57BL/6 genetic background and were 8–12 weeks old. They were fed a standard diet and water and were maintained on a 12-h light and dark cycle. The human samples were obtained at autopsy from patients who had died of acute myocardial infarction.

### Cell culture, transfection, and reagents

Primary murine VSMCs were isolated from the aortas of 4- to 6-week-old mice by enzymatic dissociation and cultured in Dulbecco's modified Eagle's medium (DMEM, Sigma, St. Louis, MO) supplemented with 10% fetal bovine serum (FBS; Hyclone, Logan, UT) and antibiotics. The VSMCs were used at passage 3–6 [13]. Small interfering RNA (siRNA) for CD44 (CD44-siRNA) and negative control siRNA (NC-siRNA) were designed at and purchased from Sigma. siRNA transfection was performed using the Lipofectamine™ RNAiMAX reagent (Invitrogen), according to the manufacturer's instructions. After 24 h of incubation, the transfection medium was removed, and the cells were then transferred to serum-free medium. HMW-HA (>950 kDa), LMW-HA (15–40 kDa), and neutralizing anti-CD44 antibody were obtained from R&D Systems. Y27632, U0126, SP600125, and SB203580 were purchased from Calbiochem or Promega Corp. All other reagents were obtained from Sigma unless otherwise specified.

### Vascular injury models

Vascular injury was caused in the mice by wire insertion or cuff placement in the right femoral artery, as described previously [14], [15]. The mice were anesthetized by intraperitoneal injection of ketamine and xylazine (100 mg/kg and 10 mg/kg, respectively), and all efforts were made to minimize suffering.

### Histological and immunohistochemical examination

The femoral arteries were embedded in OCT compound (Tissue-Tek; Miles Laboratories, IN), frozen in liquid nitrogen or fixed in zinc formalin, and embedded in paraffin. Then, 10-μm cryosections and 4-mm-thick paraffin-embedded sections were cut using a cryostat (CM-1900; Leica Microsystems GmbH, Germany). The sections were stained with hematoxylin & eosin (HE) and a biotinylated fragment of HA-binding protein (b-HABP; Seikagaku Corp., Tokyo, Japan). The b-HABP was detected using HRP-conjugated streptavidin (Vector Laboratories, Burlingame, CA). The stain was developed using the DAB substrate kit (Vector Laboratories). Neointimal formation in the femoral arteries was evaluated at 5 locations separated by a distance of 100 μm, with the most distal site located at the point where the wire-inserted branch first appeared. To quantify the intima/media (I/M) ratio, each image was digitized and analyzed under a microscope (BX-51; Olympus, Tokyo, Japan) using NIH ImageJ software. The values at 5 locations in each artery were averaged. All measurements were performed in a double-blinded manner by 2 independent researchers. Double immunofluorescence staining was performed for HA and VSMCs (Cy3-conjugated anti-αSMA; clone 1A4) or macrophages (Mac-3; clone M3/84, BD Bioscience). Cy3-labeled goat anti-rat IgG (Jackson ImmunoResearch Laboratories, Inc., West Grove, PA) and streptavidin-FITC (Vector Laboratories) were used as secondary antibodies. Immunofluorescence was observed using confocal laser scanning microscopy (Leica TCS-SP2 AOBS spectral laser scanning confocal microscopy system; Leica Microsystems) [9], [16].

### Real-time RT-PCR

Total RNA was prepared from the injured arteries or cells using ISOGEN (Nippon Gene Co., Ltd., Toyama, Japan) according to the manufacturer's instructions. Real-time RT-PCR analysis was performed using the Takara TP-800 PCR Thermal Cycler Dice Detection System (Takara Bio Inc, Shiga, Japan) to detect the mRNA expression of HAS1, HAS2, HAS3, CD44, and β-actin. The following primers were used (oligonucleotide sequences are provided in parentheses in the order of anti-sense and sense primers): HAS1, 5′-GGAGATGTGAGAATCCTTAACCCTC-3′ and 5′-TGCTGGCTCAGCCAACGAAGGAA-3′; HAS2, 5′-CCTCGGAATCACAGCTGCTTATA-3′ and 5′-CTGCCCATGACTTCACTGAAGA-3′; HAS3, 5′-GGTACCATCAGAAGTTCCTAGGCAGC-3′ and 5′-GAGGAGAATGTTCCAGATGCG-3′; CD44, 5′-GGATCCGAATTAGCTGGACACT-3′ and 5′-GCGATGCAGACGGCAAGAA-3′; and β-actin, 5′-CCTGAGCGCAAGTACTCTGTGT-3′ and 5′-GCTGATCCACATCTGCTGGAA-3′. The expression level of each target gene was normalized by subtracting the corresponding β-actin threshold cycle (CT) value; normalization was carried out using the DD C_T_ comparative method.

### Western blotting

Expression of phosphorylated extracellular-regulated kinase1/2 (p-ERK1/2), c-Jun N-terminal kinase (p-JNK), p38 (p-p38), and β-actin was analyzed by Western blotting [13]. The antibodies used in this study were purchased from Cell Signaling Technology, Inc. (Danvers, MA) or Sigma. The expression level of β-actin served as an internal control for protein loading.

### RhoA activation

RhoA GTPase activity was assessed via a pull-down assay using the RhoA Activation Assay kit (Millipore), according to the manufacturer's instructions. To confirm equal loading, total cell lysates were also subjected to direct Western blotting with an anti-RhoA antibody (Millipore).

### VSMC migration and proliferation

VSMC migration was examined using a transwell migration assay and a scratch-wound migration assay. The transwell migration assay was performed using 24-well tissue culture plates (BD Bioscience) with an 8-μm-pore polycarbonate membrane [17]. The number of migrated VSMCs was counted in 10 randomly chosen fields of duplicated chambers at a magnification of 200× for each sample. For the scratch-wound migration assay, VSMCs were scratched with a small tip along the ruler. After they were washed, the cells were cultured in serum-free DMEM for 48 h. The migration area (%) was analyzed in 10 randomly chosen fields under an inverted microscope (IX-70; Olympus) using NIH ImageJ software, and area at 0 h/area at 48 h ×100% was calculated. VSMC proliferation was determined on the basis of the uptake of 5-bromo-2′-deoxyuridine (BrdU) by using a cell proliferation ELISA kit (Roche Diagnostics, Mannheim, Germany) according to the manufacturer's instructions.

### Measurement of inflammatory cytokine levels and reactive oxygen species

The levels of interleukin (IL)-6 and monocyte chemoattractant protein-1 (MCP-1) in the culture media were assessed using the CBA Mouse Inflammation Kit (BD Biosciences) according to the manufacturer's instructions and a flow cytometer (FACSCalibur, BD Biosciences). The oxidative fluorescent dye dihydroethidium (DHE; Molecular Probes, Inc. Eugene, OR) was used to assess superoxide production as described previously. The serum levels of derivatives of reactive oxygen metabolites (d-ROMs) were measured using a Free Radical Analytic System 4 (FRAS4, H&D srl, Parma, Italy) according to the manufacturer's instructions. The d-ROMs levels are expressed in Carratelli Units (Carr U), where 1 Carr U corresponds to 0.8 mg/L of hydrogen peroxide.

### Statistical analysis

Data are expressed as mean ± SEM. The unpaired 2-tailed *t*-test was used to compare 2 groups. For comparisons between 3 or more groups, the significance of the difference between the mean values of groups was determined by one-way analysis of variance (ANOVA), followed by Tukey's multiple comparison test. All analyses were performed using Prism5 (GraphPad Software Inc., San Diego, CA). *p*<0.05 was considered statistically significant.

## Results

### HA in neointimal formation after vascular injury

**Figure 2:**
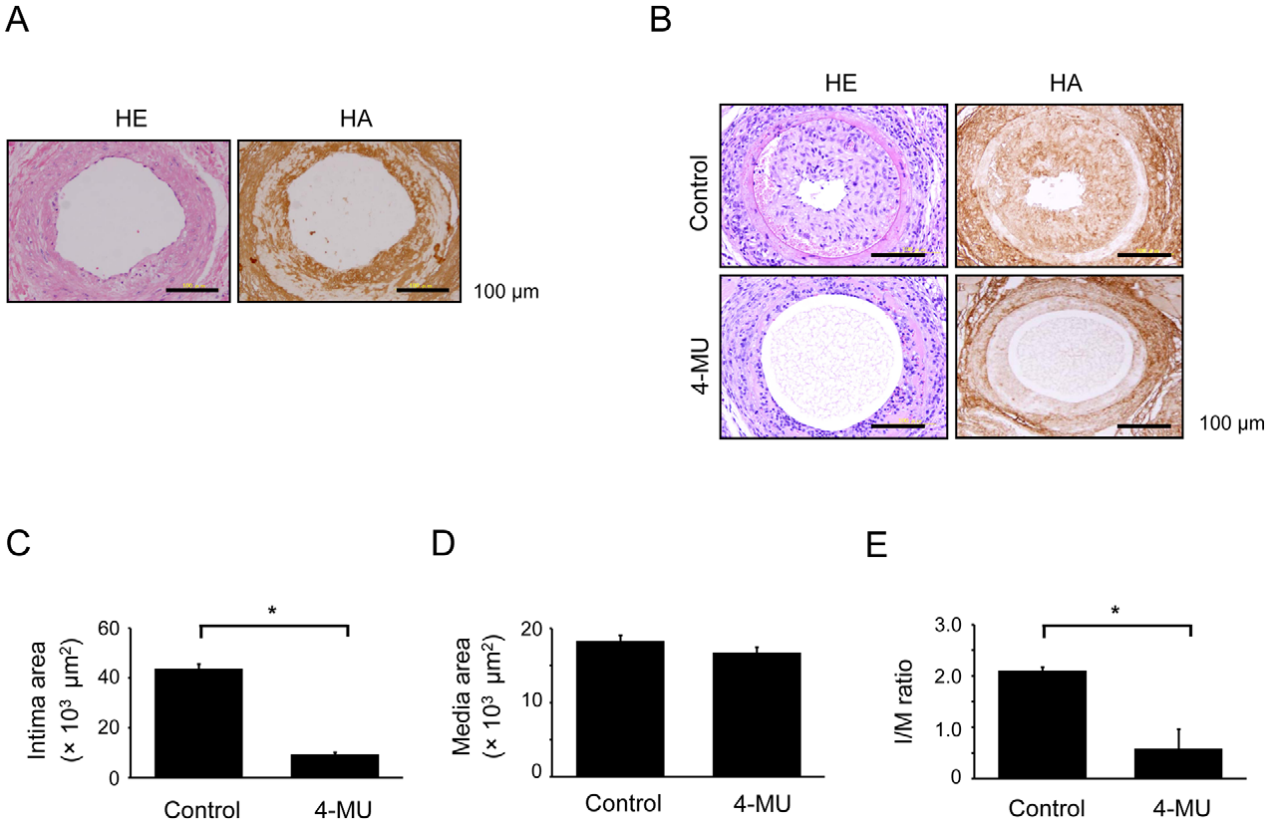
HA in human atherosclerosis and neointimal formation after murine vascular injury. (A) HE staining and staining for HA detection in human atherosclerotic plaques from the coronary artery. (B–E) Mice were orally treated with vehicle or 4-MU (6 mg·g^−1^·d^−1^) 3 days before wire-mediated vascular injury, and the injured arteries were excised at 21 days after injury. The sections were stained with HE and for HA (B), and neointimal formation was evaluated. The bar graphs show the area of the neointimal region (C), area of the medial region (D), and I/M ratio (E). Arrowheads indicate the borders of the media. Data are mean ± SEM (n = 20 [mouse number] for each, *p<0.001).

We first investigated whether HA was expressed in neointimal lesions of human coronary atherosclerotic plaques. Consistent with a previous report [18], HA was clearly visible in the neointimal lesions, but it not clearly detectable in the medial area (Fig. 2A). To determine whether HA is critical for neointimal formation, we produced a murine model of wire-mediated vascular injury models. Since we previously demonstrated that neointimal formation is completed at 21 days in wire-mediated vascular injury [16], [19], we evaluated the role of HA at this point. Similar to the expression pattern in human atherosclerotic plaques, HA was markedly expressed in the neointimal area, whereas it was not clearly detectable in the medial area (Fig. 2B). Treatment with a pharmacological inhibitor of HA synthesis (4-MU; 6 mg·g^–1^·d^–1^ [20], [21]) almost completely inhibited neointimal formation after the injury (Fig. 2B). Quantitative analysis showed that in 4-MU-treated mice, the neointimal area and intima/media (I/M) ratio were significantly reduced at 21 days after the injury (Figs. 2C–E). No significant difference was observed in the medial area between the vehicle- and 4-MU-treated mice. No significant differences in blood cell counts and body weights were found between vehicle- and 4-MU-treated mice (data not shown).

### HA induces migration and Rho activation via CD44 *in vitro*

**Figure 3:**
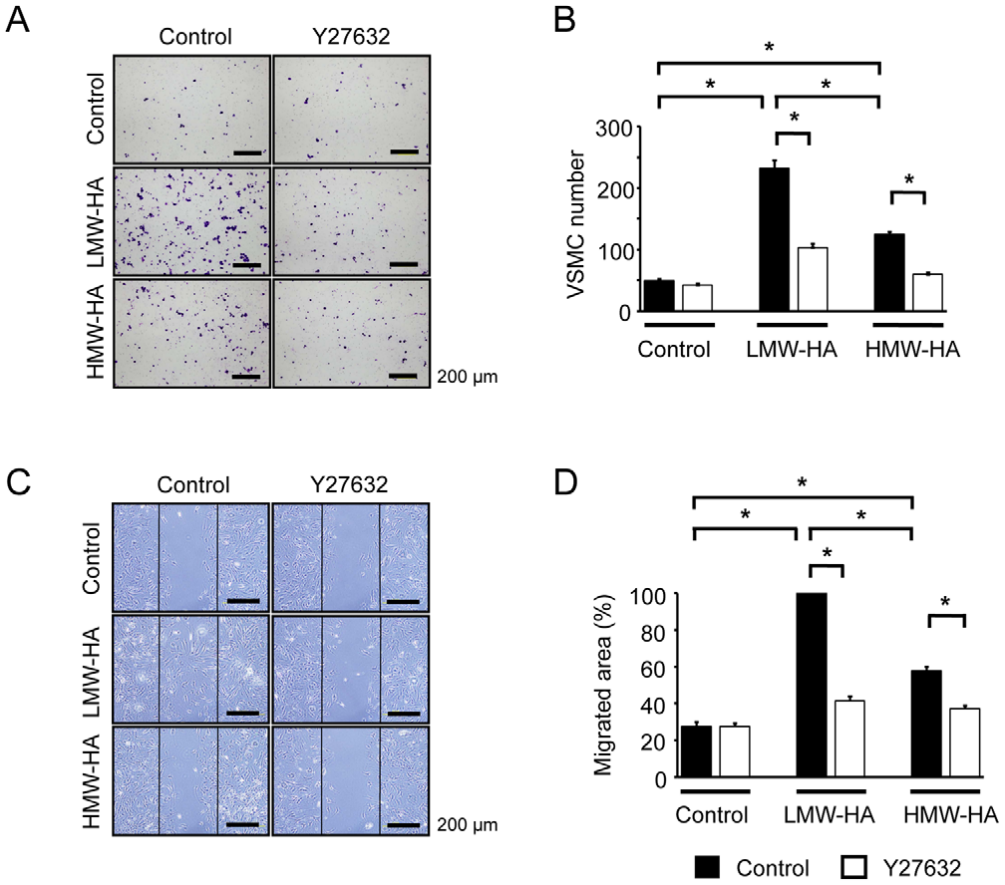
Role of Rho kinase in HA-induced VSMC migration. (A–D) Primary VSMCs were pretreated with vehicle or Y27632 (Rho kinase inhibitor) for 1 h, and VSMC migration in response to LMW-HA or HMW-HA (1 mg/mL) was then measured using a modified Boyden chamber transwell migration assay (6 h, A and B) and scratch-wound migration assay (48 h, C and D). Data are mean ± SEM (n = 10 for each, *p<0.001).

VSMCs are the main cellular components of neointimal lesions after vascular injury, and their migration and proliferation can contribute to the development of these lesions. Further, HA was found to be expressed in VSMCs of the neointima (Fig. S1). Therefore, the effect of HA on VSMC behavior was investigated *in vitro* using primary VSMCs isolated from murine aortas. The biological effects of HA have been shown to depend on its molecular chain length [5]; therefore, the cells were stimulated with both LMW-HA and HMW-HA. A transwell migration assay showed that LMW-HA significantly accelerated VSMC migration (Figs. 3A and B). HMW-HA also increased migration but was less effective than LMW-HA. In addition, the VSMC migration induced by LMW-HA and HMW-HA was significantly inhibited by treatment with the Rho kinase inhibitor Y27632 (Figs. 3A and B). To confirm these findings, a scratch-wound migration assay was performed, and it yielded similar results, i.e., LMW-HA significantly stimulated VSMC migration, and Y27632 inhibited this migration (Figs. 3C and D).

**Figure 4:**
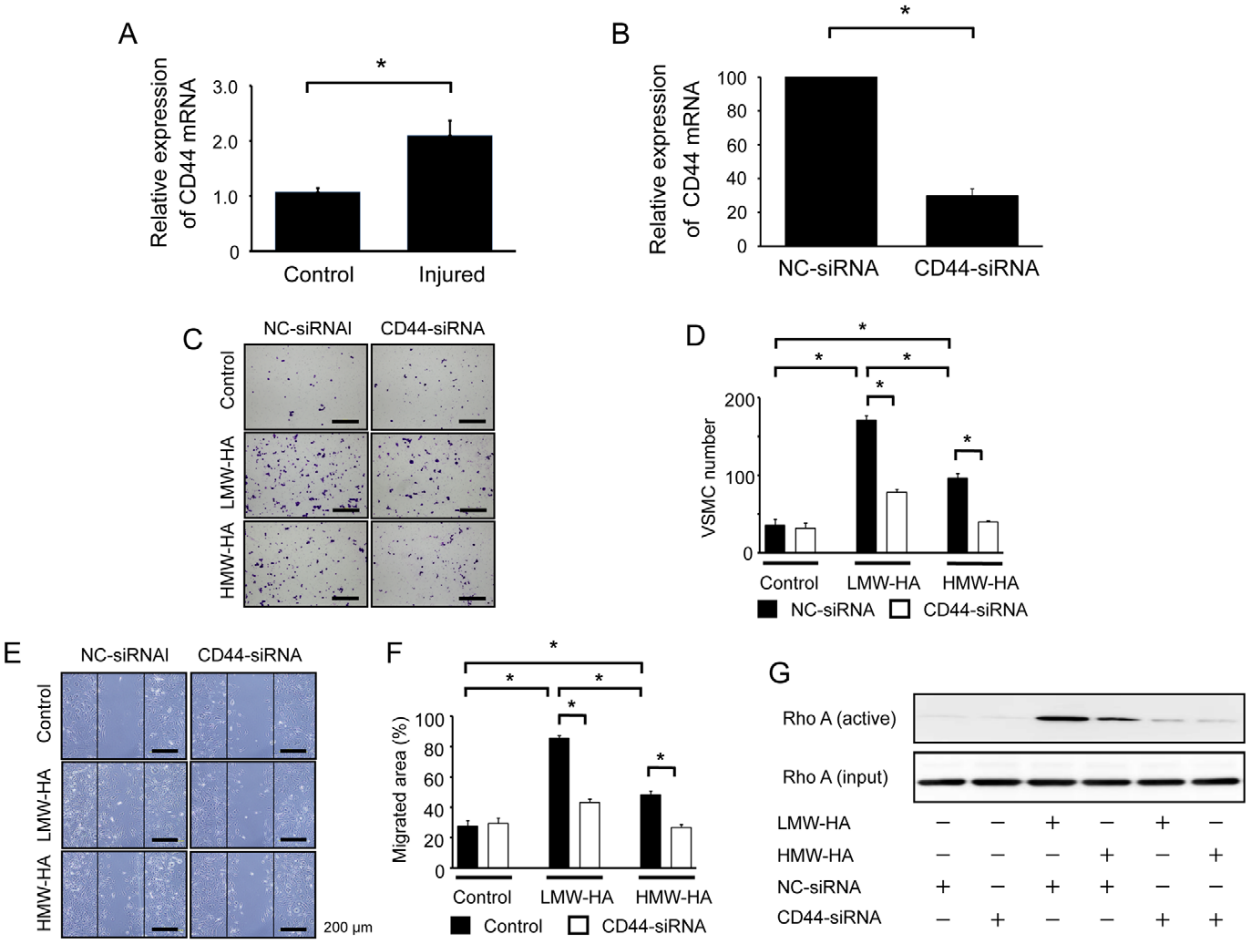
Role of CD44 in HA-induced VSMC migration. (A) Total RNA was extracted from the injured arteries at 21 days after vascular injury and analyzed for mRNA expression of CD44 by real-time RT-PCR. Data are expressed as mean ± SEM (n = 10 for each, *p<0.001). (B) After VSMCs were transfected with either CD44-siRNA or NC-siRNA, total RNA was extracted from the cells and analyzed for CD44 mRNA expression by real-time RT-PCR. Data are expressed as mean ± SEM (n = 8 for each, *p<0.001). (C–F) VSMCs were transfected with either NC- or CD44-targeted siRNA for 48 h, and their migration in response to LMW-HA or HMW-HA (1 mg/mL) was measured using a modified Boyden chamber transwell migration assay (6 h, C and D) and scratch-wound migration assay (48 h, E and F) Data are mean ± SEM (n = 10 for each, *p<0.001). (G) VSMCs were transfected with either NC- or CD44-targeted siRNA for 48 h and then stimulated with LMW-HA or HMW-HA for 60 min. Cell lysates were analyzed by Western blotting with antibodies against activated RhoA. The results are representative of 3 independent experiments.

**Figure 5:**
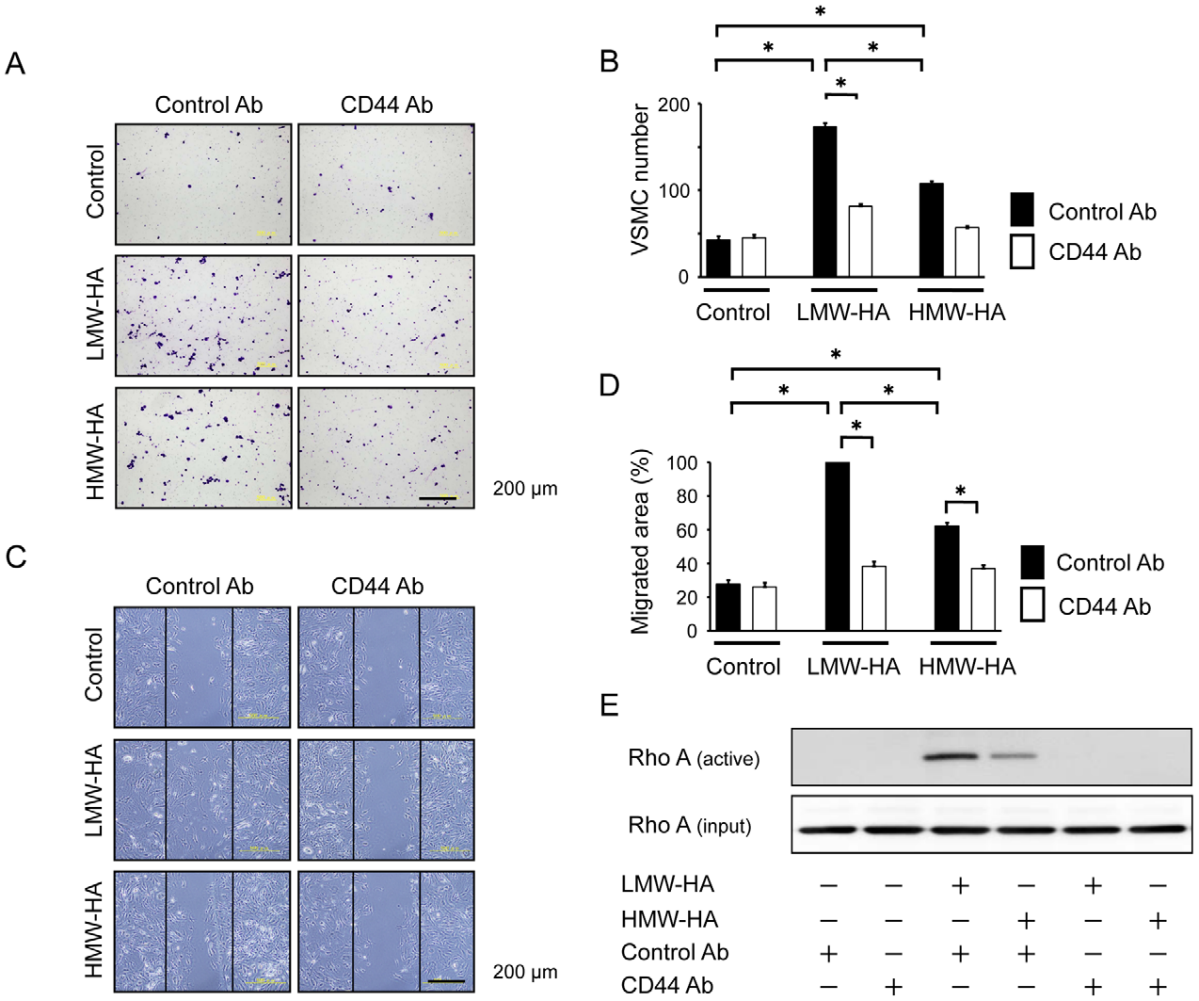
Effect of anti-CD44 antibody on HA-induced VSMC migration. VSMCs were pretreated with either control IgG or neutralizing anti-CD44 antibody (5 μg/mL) for 6 h and then VSMC migration in response to LMW-HA or HMW-HA (1 mg/mL) was then measured using a modified Boyden chamber transwell migration assay (6 h, A and B) and scratch-wound migration assay (48 h, C and D). Data are mean ± SEM (n = 8 for each, *p<0.001). (E) VSMCs were pretreated with control IgG or neutralizing anti-CD44 antibody (5 μg/mL) for 6 h and then stimulated with LMW-HA or HMW-HA for 60 min. Cell lysates were analyzed by western blotting with antibodies against activated RhoA. The results are representative of 3 independent experiments.

CD44 is considered an important receptor for HA in a wide variety of cells, including VSMCs [22], [23], We observed that CD44 mRNA expression was increased in the injured arteries compared to the controls (Fig. 4A). In addition, treatment with LMW-HA and HMW-HA increased CD44 mRNA expression in VSMCs (Fig. S2). To address the role of CD44 in VSMC migration, siRNA was used to downregulate CD44 expression (Fig. 4B). CD44-siRNA, but not NC-siRNA, significantly decreased LMW- or HMW-HA-induced VSMC migration, as determined by both a transwell migration assay (Figs. 4C and D) and a scratch-wound migration assay (Figs. 4E and F). Since the Rho kinase inhibitor Y27632 also inhibits VSMC migration, we investigated whether the small G-protein Rho can be a downstream effector of the HA-CD44 interaction. LMW-HA clearly stimulated Rho activation in VSMCs as did HMW-HA. Further, RhoA activation was almost completely inhibited by CD44-siRNA (Fig. 4G). To further confirm the role of CD44, CD44 was blocked with its neutralizing antibody, and this was found to inhibit VSMC migration (a transwell migration and scratch-wound migration assays) and RhoA activation (Fig. 5).

### HA induces proliferation and activates mitogen-activated protein (MAP) kinases

**Figure 6:**
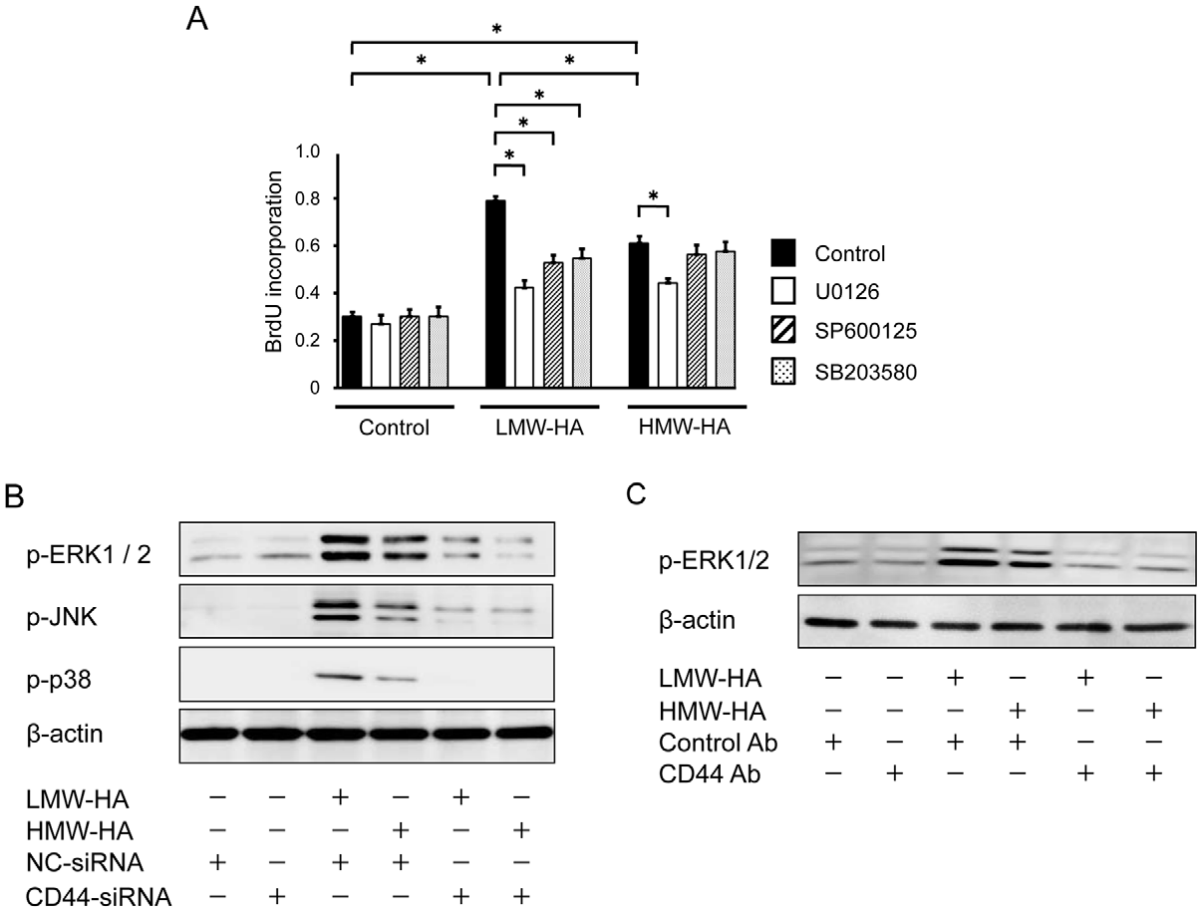
HA induces proliferation and MAP kinase activation. (A) Primary VSMCs were pretreated with vehicle, U0126 (ERK inhibitor), SP600125 (JNK inhibitor), or SB203580 (p38 inhibitor) for 1 h, and the proliferation of VSMCs in response to LMW-HA or HMW-HA (1 mg/mL) was measured using a BrdU incorporation assay. Data are expressed as mean ± SEM (n = 10 for each, *p<0.001). (B) VSMCs were transfected with either NC- or CD44-targeted siRNA for 48 h and then stimulated with LMW-HA or HMW-HA for 60 min. Cell lysates were analyzed by Western blotting with antibodies against p-ERK1/2, p-JNK, p-p38, and β-actin. The results are representative of 3 independent experiments. (C) Effect of anti-CD44 antibody on ERK1/2 activation. VSMCs were pretreated with control IgG or neutralizing anti-CD44 antibody (5 μg/mL) for 6 h and then stimulated with LMW-HA or HMW-HA for 20 min. Cell lysates were analyzed by Western blotting with antibodies against p-ERK1/2 and β-actin. The results are representative of 3 independent experiments.

The effect of HA on VSMC proliferation was explored using a BrdU incorporation assay. LMW-HA significantly stimulated the proliferative activity of VSMCs (Fig. 6A), as did HMW-HA. LMW-HA-stimulated VSMC proliferation was significantly inhibited by treatment with MAP kinase inhibitors (U0126 for ERK1/2, SP600125 for JNK, and SB203580 for p38) (Fig. 6A). On the other hand, HMW-HA-stimulated VSMC proliferation was significantly inhibited by U0126 but not by SP600125 and SB203580. Western blot analysis showed that both LMW-HA and HMW-HA activated ERK1/2, JNK, and p38, and as expected, this activation was completely inhibited by treatment with the corresponding inhibitors of the MAP kinases (data not shown). Further, HA-stimulated activation of ERK1/2 was inhibited by CD44-siRNA or CD44 neutralizing antibody (Figs. 6B and C).

### HA stimulates inflammatory cytokine and ROS production

**Figure 7:**
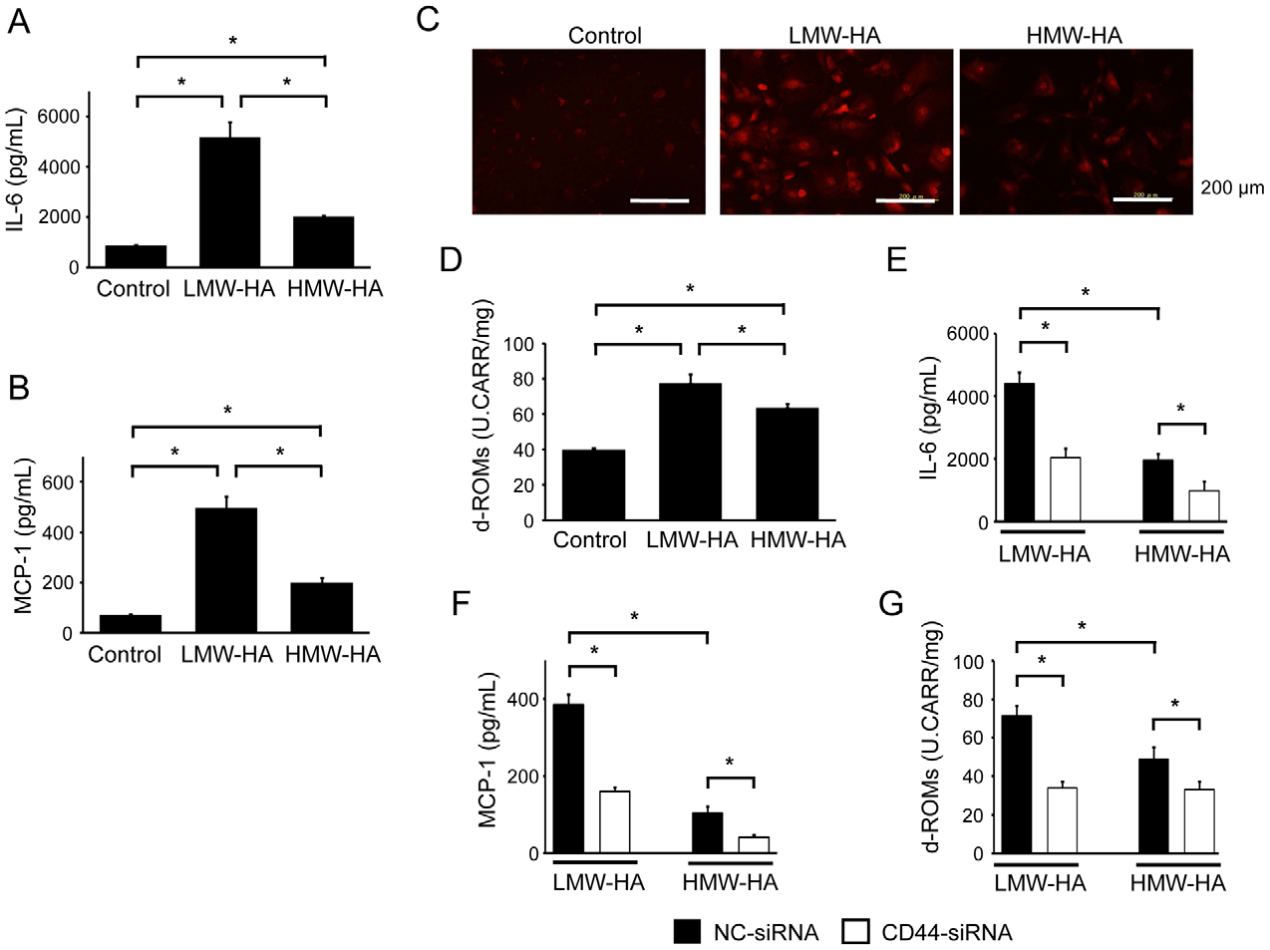
HA stimulates inflammatory cytokine and ROS production. (A and B) Primary VSMCs were stimulated with LMW-HA or HMW-HA for 24 h. The levels of IL-6 (A) and MCP-1 (B) in the supernatants were assessed. Data are expressed as mean ± SEM (n = 10 for each, *p<0.001). (C) VSMCs were stimulated with LMW-HA or HMW-HA for 24 h and stained with DHE. The results are representative of 3 independent experiments. (D) VSMCs were stimulated with LMW-HA or HMW-HA for 24 h. The levels of d-ROM in the supernatants were assessed. Data are expressed as mean ± SEM (n = 10 for each, *p<0.001). (E–G) VSMCs were transfected with either NC- or CD44-targeted siRNA for 48 h and then stimulated with LMW-HA or HMW-HA for 24 h. The levels of IL-6 (E), MCP-1 (F), and d-ROMs (G) in the supernatants were assessed. Data are expressed as mean ± SEM (n = 10 for each, *p<0.001).

Accumulating evidence has indicated the importance of inflammatory cytokines and ROS in the process of neointimal formation after vascular injury [24]. Here, IL-6 and MCP-1 production in the culture media of VSMCs was significantly increased by LMW-HA (Figs. 7A and B). In addition, LMW-HA-induced ROS production was confirmed by DHE staining and a d-ROMs test (Figs. 7C and D). HMW-HA had similar effects on the production of IL-6, MCP-1, and ROS; it was less effective effects than LMW-HA. Further, the production of these cytokines and ROS was significantly inhibited by siRNA-mediated CD44 downregulation (Figs. 7E–G).

### HA overexpression in VSMCs enhances neointimal formation after vascular injury

**Figure 8:**
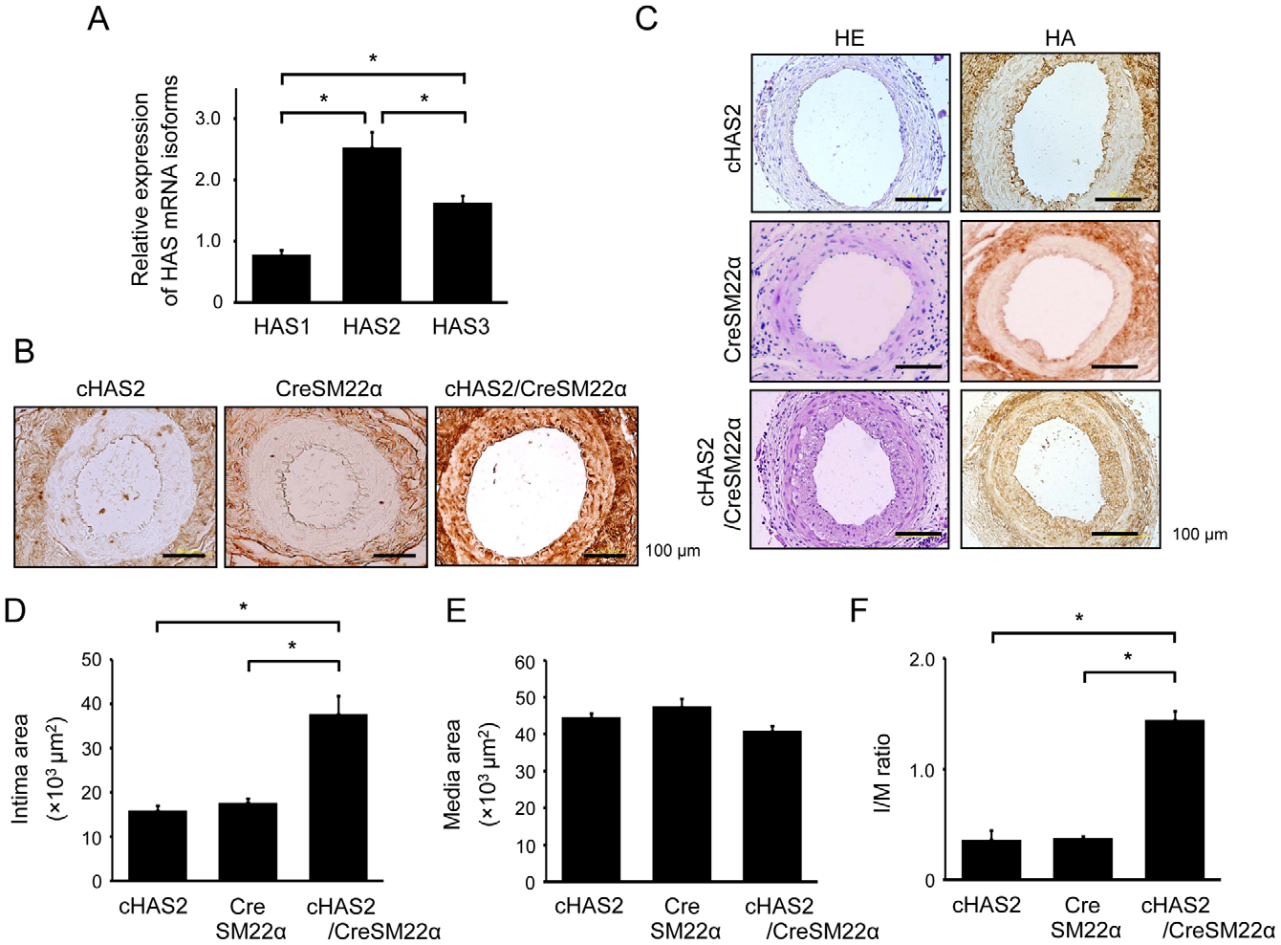
HA overexpression in VSMCs enhances neointimal formation. (A) Total RNA was extracted from the injured arteries at 21 days after vascular injury and analyzed for the mRNA expression of HAS1, HAS2, and HAS3 using real-time RT-PCR. Data are expressed as mean ± SEM (n = 10 for each, *p<0.001). (B) Intact arteries were excised from cHAS2, CreSM22α, or cHAS2/CreSM22α mice. The sections were stained with HA. (C–F) The injured arteries were excised from cHAS2, CreSM22α, or cHAS2/CreSM22α mice at 21 days after cuff-mediated vascular injury. The sections were stained with HE and for HA (C), and neointimal formation was evaluated. The bar graphs show the area of the neointimal region (D), the area of the medial region (E), and I/M ratio (F). Data are mean ± SEM (n = 10, *p<0.001).

To further investigate the cell-specific role of HA in vascular injury, a genetic strategy was employed to specifically overexpress HAS2 in VSMCs (cHAS2/CreSM22α), since HAS2 is the most prominent enzyme of the 3 HAS isoforms at the site of vascular injury (Fig. 8A). After increased HA expression in the medial area of the intact artery was confirmed in cHAS2/CreSM22a mice (Fig. 8B), a cuff placement-mediated vascular injury model was produced on the basis of the hypothesis that HAS2 overexpression can accelerate neointimal formation. Consistent with previous reports [15], neointimal formation in the control mice (cHAS2 and CreSM22a mice) was modest at 21 days after the injury (Fig. 8C). In contrast, robust neointimal formation was detected in the injured arteries of cHAS2/CreSM22a mice. Quantitative analysis showed that the neointimal area and the I/M ratio of cHAS2/CreSM22a mice were significantly increased (Figs. 8D–F). No significant difference was observed in the medial area among the 3 groups.

### Effects of HAS2 overexpression on VSMC behavior *in vitro*

**Figure 9:**
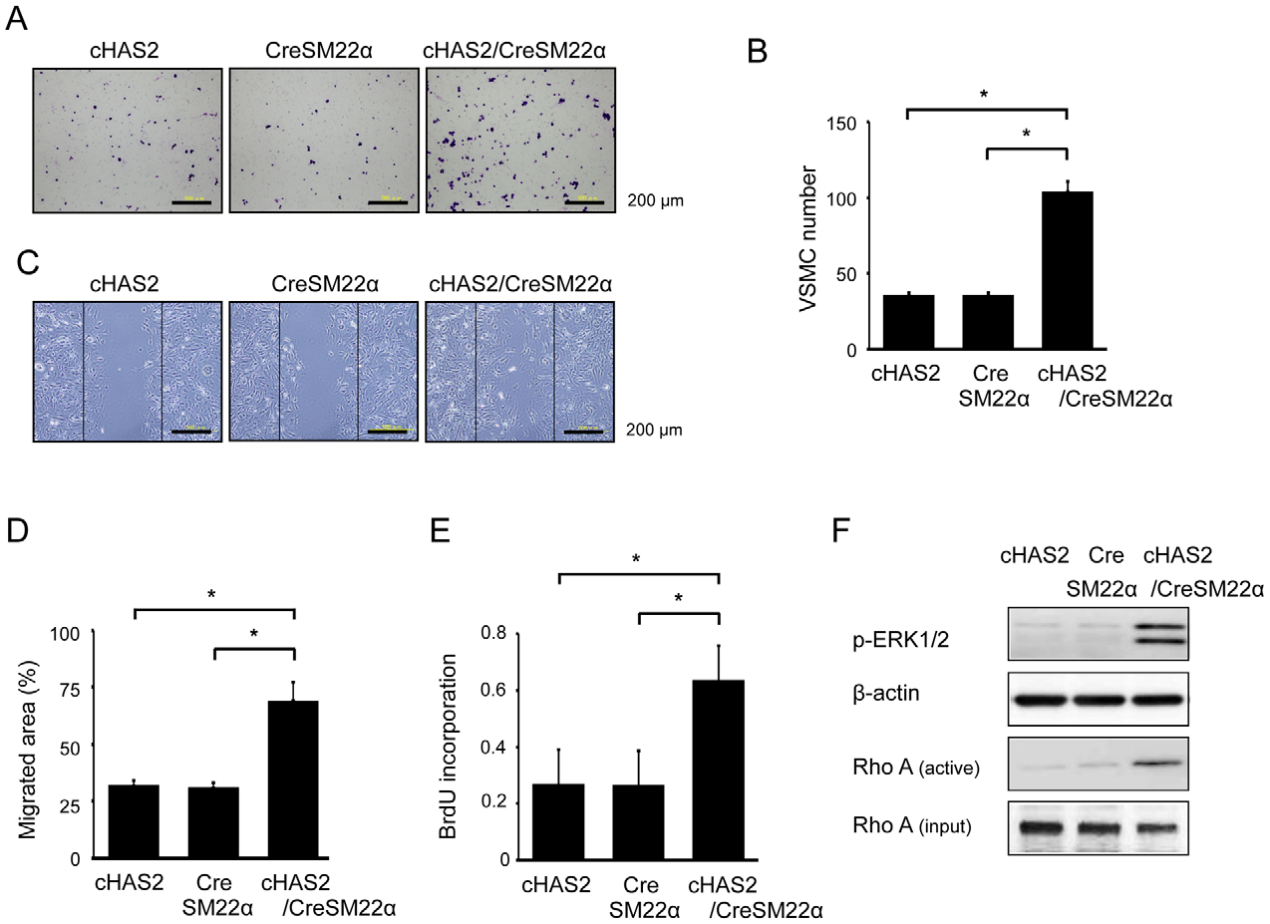
Effects of HAS2 overexpression on VSMC behavior. Primary VSMCs were prepared from cHAS2, CreSM22α, or cHAS2/CreSM22α mice. (A–D) Spontaneous VSMC migration was measured using a modified Boyden chamber transwell migration assay (A and B, 6 h, n = 10 each, *p<0.001) and scratch-wound migration assay (C and D, 48 h, n = 10 for each, *p<0.001). (E) VSMC proliferation activity was measured using a BrdU incorporation assay. Data are expressed as mean ± SEM (n = 10 for each, *p<0.001). (F) Cell lysates were prepared from VSMCs and analyzed by Western blotting with antibodies against p-ERK1/2 and β-actin. RhoA activation was assessed using a pull-down assay. The results are representative of 3 independent experiments.

**Figure 10:**
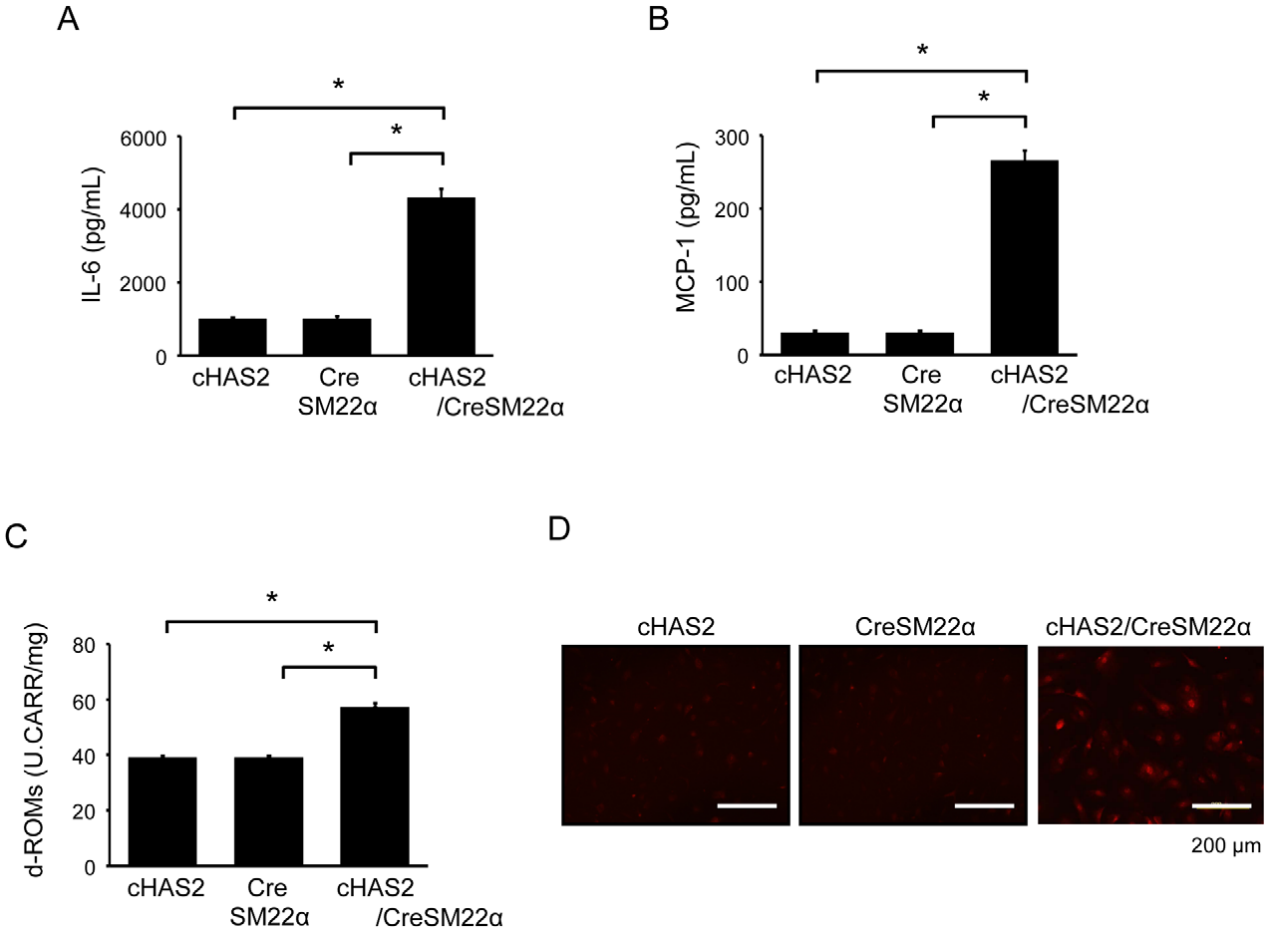
Production of cytokines and ROS in HA-overexpressing VSMCs. (A–D) Production of cytokines and ROS in HA-overexpressing SMCs. VSMCs were prepared from cHAS2, CreSM22α, or cHAS2/CreSM22α mice. Cells were cultured for 24 h, and the levels of IL-6 (A), MCP-1 (B), and d-ROMs (C) in the supernatants were assessed. Data are expressed as mean ± SEM (n = 10 for each, *p<0.001). Cells were also stained with DHE (D). The results are representative of 3 independent experiments.

To examine VSMC behavior in cHAS2/CreSM22α mice, primary VSMCs were prepared from murine aortas and used in the following experiments. HA-overexpressing VSMCs showed a significant increase in migration activity (Figs. 9A–D), proliferation activity (Fig. 9E), and ERK1/2 and RhoA activation (Fig. 9F), although no such ERK1/2 and RhoA activation was observed in control VSMCs from cHAS2 and CreSM22α mice. HA overexpression also increased the production of IL-6, MCP-1, and ROS (Figs. 10A–D). Further, CD44 mRNA expression was increased in HA-overexpressing VSMCs (Fig. S3).

## Discussion

The major findings of this study are as follows: (1) HA was markedly expressed in human atherosclerotic plaques and murine neointimal lesions after vascular injury. (2) The pharmacological inhibition of HA synthesis by 4-MU attenuated neointimal formation after wire-mediated vascular injury. (3) LMW-HA accelerated VSMC migration by activating the CD44/Rho kinase pathway, and (4) it promoted VSMC proliferation through the CD44/ERK1/2 pathway. (5) LMW-HA also stimulated the production of IL-6, MCP-1, and ROS. (6) HAS2 was predominantly expressed in the injured arteries, and cTg mice overexpressing the *HAS2* gene specifically in VSMCs showed accelerated neointimal formation after cuff-mediated vascular injury. (7) Lastly, HAS2-overexpressing VSMCs showed augmented migration and proliferation as well as inflammatory cytokines and ROS production. Collectively, these findings suggest that VSMC-derived HA plays an important role in the progression of neointimal formation after vascular injury.

HA is a major component of the ECM. It has been shown to exert distinct biological effects depending on the isoform, i.e., the molecular weight. In mammals, HAS1 and HAS2 produce HMW-HA, whereas HAS3 produces LMW-HA [3], [4]. HMW-HA is the predominant isoform under physiological conditions. However, it fragments during inflammation and tissue injury, and these HA fragments (i.e., LMW-HA) induce the expression of inflammatory cytokines and enhanced the release of ROS, leading to enhanced inflammatory responses and tissue damage. In the present study, we showed that LMW-HA was much more effective than HMW-HA in influencing VSMC behavior, including enhanced migration, proliferation, and the production of cytokines and ROS. Further, this VSMC behavior promoted by LMW-HA was mediated by CD44. Several studies have reported that CD44 is the main receptor for HA in VSMCs [22]. One study found that CD44 deficiency reduced the size of atherosclerotic plaques in apoE^–/–^ mice, which occurred partly because of impaired VSMC migration and proliferation [23], [25]. We further demonstrated that HA-induced migration and proliferation were mediated by the RhoA-CD44 and ERK1/2-CD44 pathways, respectively. Another HA receptor, receptor for HA-mediated mobility (RHAMM), has also been reported to play a role in VSMC behavior [4]. Savani et al. [26] showed that RHAMM is necessary for the bovine VSMC migration after wounding injury. In addition, Goueffic et al. [27] demonstrated HA induces VSMC migration through RHAMM-mediated Rac activation. RHAMM has been found on cell surfaces but is also present in the cytosol and nucleus. RHAMM and CD44 seem to have overlapping functions in their influence on VSMC behavior. Further investigation is needed to understand the precise signaling pathways of HA-regulated VSMC behavior.

Several reports have described the role of HA in the development of atherosclerosis. Chai and colleagues [6] generated mice overexpressing human HAS2 under the control of the αSMA promoter, and crossed these mice with apoE^–/–^ mice, and showed that overproduction of HA in the aorta of apoE^–/–^ mice accelerated the development of atherosclerosis. Riessen et al. [8] showed that HA production in neointimal lesions was increased in balloon-injured rat carotid arteries as well as in human restenotic lesions after PCI and found that HA production corresponded with VSMC proliferation. This report suggests a causal association between HA and VSMC proliferation. Moreover, Jain et al. [28] showed that HA and CD44 expression was increased in balloon-injured rat arteries and that HA-induced DNA synthesis in cultured VSMCs was mediated by CD44. However, the exact role played by HA in vascular injury remains to be determined. We observed marked production of HA in the neointimal lesions after vascular injury. Importantly, the inhibition of HA production significantly reduced neointimal formation, whereas HA overproduction in VSMCs promoted neointimal formation. Taken together, our data indicate that vascular injury induces HA production in migrating and proliferating VSMCs, thereby resulting in accelerated neointimal formation.

In the present study, we used the pharmacological agent 4-MU to inhibit HA synthesis and showed that 4-MU treatment considerably attenuated neointimal formation after vascular injury. In this regard, we observed 4-MU inhibited HA production without effect on proliferation in VSMCs *in vitro*, (Figure S4) supporting that the inhibitory effect of 4-MU on intimal formation *in vivo* might be mediated by decreased HA production. Although the bioavailability of 4-MU is unclear, it has been reported that oral administration of 4-MU in mice reduced the HA content in the liver, but not in the skin, lungs, and brains [20]. The inhibitory effect of 4-MU is mainly due to the downregulation of HAS and depletion of the HA precursor UDP-GlcUA by activation of UDP-glucuronyl transferases [29]. In particular, 4-MU is most effective against HAS2 [30], which is predominantly expressed in injured arteries. Surprisingly, Nagy et al. [7] recently showed that 4-MU treatment for 21 weeks in Western diet-fed apoE^–/–^ mice resulted in accelerated atherosclerosis. They showed that 4-MU severely damaged the endothelial glycocalyx, which is a vasoprotective barrier for endothelial cells, and therefore promoted inflammatory and thrombotic responses in the vascular wall, proposing this as a possible mechanism of action of 4-MU. There are several reasons for the difference observed in the effects of 4-MU between Nagy's study and the present study. First, the endothelial glycocalyx is not involved in the vascular injury model used in our study because endothelial cells were completely denuded in this model [19]. This endothelial denudation may effectively inhibit HA production in VSMCs. Indeed, insufficient inhibition of HA production was observed in the atherosclerotic plaques in Nagy's study. Second, since neointimal lesions after vascular injury are mainly composed of VSMCs [31]. the inhibitory effect of 4-MU on HA synthesis might depend on the types of cells and tissues. The safety of 4-MU therapy in humans has been already demonstrated and it is used clinically for the treatment of hepatobiliary diseases [32], [33]. In addition, a clinical trial of 4-MU in chronic hepatitis B and C is also currently underway (ClinicalTrial.gov: NCT00225537). However, further investigations to understand the precise action of 4-MU are needed to use vascular diseases including atherosclerosis and vascular injury.

In conclusion, we clearly demonstrated the importance of HA in the development of neointimal formation by using 2 types of vascular injury models. In particular, the specific overexpression of HA in VSMCs promoted the development of neointimal formation. Further, *in vitro* experiments revealed that LMW-HA enhanced migration, proliferation, and inflammatory cytokine and ROS production in VSMCs via a CD44-dependent mechanism. Since vascular injury is an initiating event in various types of cardiovascular diseases, these findings suggest that HA could be a potential therapeutic target for cardiovascular disease.

## Supporting Information

Figure S1 **HA expression in neointimal VSMCs.** Wire-mediated vascular injury was produced in wild-type mice, and the injured arteries were excised at 21 days after injury. Double immunofluorescent staining for HA and αSMA or Mac-3 was performed.
(TIF)

Figure S2 **Effects of LMW-HA and HMW-HA on CD44 expression in VSMCs.** VSMCs were stimulated with LMW-HA and HMW-HA, and the CD44 mRNA expression was analyzed by real-time RT-PCR. Data are expressed as mean ± SEM (n = 10 for each, *p<0.001).
(TIF)

Figure S3 **CD44 mRNA expression in HA-overexpressing VSMCs.** VSMCs were prepared from cHAS2, CreSM22α, or cHAS2/CreSM22α mice. Total RNA was extracted and analyzed for CD44 mRNA expression using real-time RT-PCR. Data are expressed as mean ± SEM (n = 8 for each, *p<0.001).
(TIF)

Figure S4 **Effects of 4-MU on HA production and proliferation in VSMCs.** VSMCs were treated with 4-MU (0.2 and 0.5 mM) for 24 h. (A) HA production in the supernatants were assessed. (B) VSMC proliferation activity was measured using a BrdU incorporation assay. Data are expressed as mean ± SEM (n = 8 for each, *p<0.05).
(TIF)
