# Supplementary figures and images for: Crucial Role of Hyaluronan in Neointimal Formation after Vascular Injury
Source: PLoS One. 2013 Mar 6;8(3):e58760. doi: 10.1371/journal.pone.0058760 (PMC3590137; doi:10.1371/journal.pone.0058760)

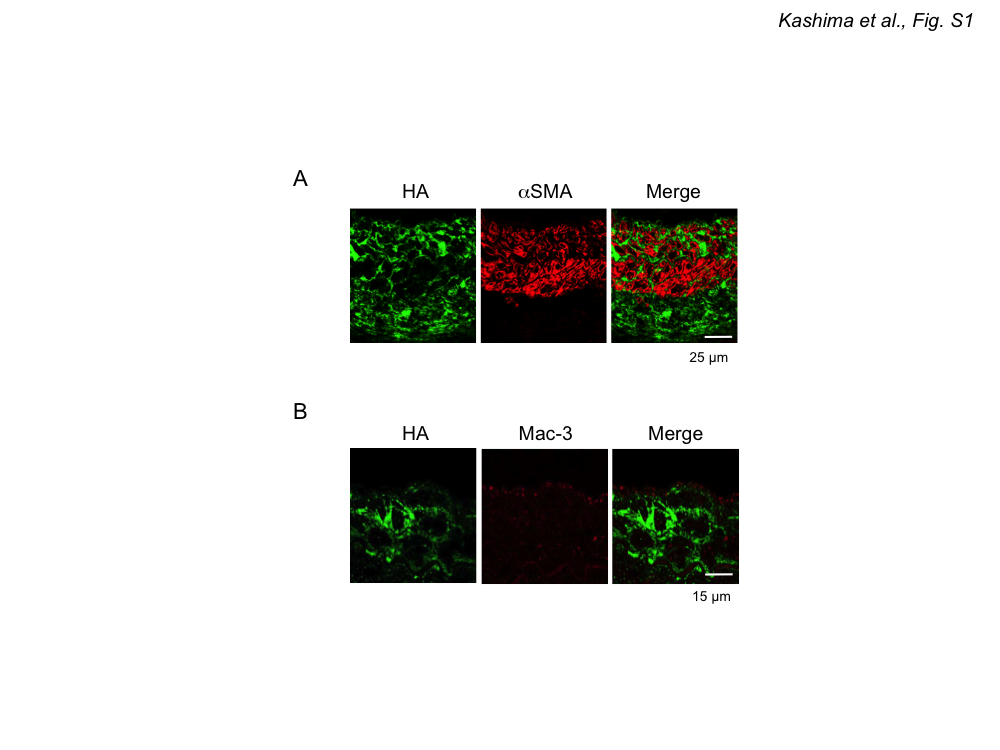

Supplement: Figure S1 — HA expression in neointimal VSMCs. Wire-mediated vascular injury was produced in wild-type mice, and the injured arteries were excised at 21 days after injury. Double immunofluorescent staining for HA and αSMA or Mac-3 was performed. (TIF) [file pone.0058760.s001.tif]
